
JOURNAL INFORMATION
==============================
NLM Title Abbreviation: Atlanta Med Surg J (1884)
NLM Journal ID: 101251623

Atlanta Medical and Surgical Journal (1884)

Publisher: Biomedical Journal Digitization Project

ARTICLE INFORMATION
==============================
PMCID: PMC9199303
PMID: 35829596
Article version: 1
Subjects: Original Communications

Vaccination and Its Sores* Remarks before the Atlanta Society of Medicine, March 17, 1893, with discussion.

Hutchins M. B. M.D. Atlanta, Ga.

Print publication date: 1898 Dec
Volume: 15
Volume Series: New Ser
Issue: 10
First page: 658
Last page: 666
License: This work is free of known copyright restrictions. For more information, please see PMC Back Issue Digitization.
License URL: https://creativecommons.org/publicdomain/mark/1.0/

==============================

### VACCINATION AND ITS SORES.*

By M. B. HUTCHINS, M.D.,

ATLANTA, GA.

I should like, first, to say something about the method which I showed the Society last fall of vaccination without an instrument, the so-called painless method. The only objection to it is that it is rather slow; and I found that I was failing in a good many cases, failing more than I should and more than other people who were trying the method. I couldn't account for it until I received a circular from one of the vaccine farms saying that you should not wet the points too much. Then I got better results. Some time after that demonstration was made, I had a case in the office to vaccinate and I had liquor potassæ, and I went back on what I said about a stick of caustic potash. You know when you take it out of the bottle it begins to take on moisture. Anyway, I took the stick of caustic potash and as soon as a little moisture began forming, I touched one or two spots with this liquid, and smoothed it a little bit. I immediately took a little piece of damp cotton, and with that method I succeeded very rapidly and well, more rapidly than in any of the old ways. Where you wipe off the deliquesced potash immediately, there is no pain and no injury to the deeper tissues. One of my doctor friends told me one night that I had not put enough on him. So I put more to satisfy him, and now he has a nice hypertrophied scar as a result; and he wanted some more.

You gentlemen, I suspect, have had more experience with vaccination than I have had, but I have had some experience with these old sores that are left after vaccination. I will say first that I believe as soon as the patient has had slight fever and the inflammatory action, which so frequently takes place and the redness begins to subside, that the vaccination has done its work, and consequently if there is a great deal of inflammation and a great deal of suppuration under the scab, I think it is proper to remove the scab and clean the sore and heal it. I had to treat a little boy, and I have

* Remarks before the Atlanta Society of Medicine, March 17, 1893, with discussion.

seen several other cases, where the arm or leg had been neglected for eight weeks or some months. I don't think it is at all necessary for the sore to be allowed to run its course. You can save the patient a great deal of discomfort by not doing so. I saw an old woman the other day and examined her arm and there was an old scab there that she said had been there for about eight weeks.

The patient may scratch or rub it, or the clothing rub it. And these vaccination shields sold in drug stores, in my opinion, do more harm than good. For instance, at the time that you need it worst the whole area around is very much inflamed. Put on one of these shields, and as likely as not you will find it down, perhaps, over the sore the next time that you look for it. Dr. Hurt told me that he had a beautiful sore arm on a patient where the shield had slipped down, inoculating as it went. Another thing is, these vaccination sores are kept up by constant infection and reinfection. Some of them are not genuine vaccination sores at all, but simply infected ones. In some cases they are caused by some organic change in the virus on the point, so that there was not a typical result, and in some other cases I have found that the harm resulted from people trying to treat themselves. They don't all have the same symptoms. In some cases they have got something on that they can't get off, and in some other cases they get it off too often.

As regards the treatment of these sores, I do not think there is any doubt about the propriety of treating them as soon as the patient has passed the acute stage of vaccination. I don't pretend to try to tell you gentlemen how it should be done further than to give my little experience, and to call your attention to the general principles underlying the treatment of ulcers. As a rule, you see these cases in one of two or three forms. One of the most constant probably is an old ulcer full of pus, covered over with a dirty brown scab. In other cases you will see that the scab has been kept off a little too well and you have a lesion there composed of fungating granulations. Sometimes that is all the matter; there is just enough exudation to give you a crust on it and give it the appearance of a sore, and it goes on that way for months and months. There is another thing you will sometimes see in these sores. That is, owing to the influence of opposing organisms, you get a coagulation over these ulcers of a dead membranous substance which interferes with the formation of a new epidermis and cicatrization. In the same way you get something very much like coagulation—necrosis from the use of strong antiseptics in the treatment of these ulcers or sores. You get a coagulation there, a something that interferes as much as possible with the healing of these sores. I am reminded here of an ulcer on the ankle that I treated a few years ago, where I used ichthyol until I had it just a coagulated membrane. As to the method, there is little to say. I will just give you my way.

The first thing is to remove the crust, and this is often very easy, because it is hanging by the slightest attachment. If you cannot get rid of it by just lifting it off, it can be softened up usually with some antiseptic oil. Then the sore is cleaned, usually with the bichloride of mercury solution. I have found in the treatment of ulcers that it is better not to use hydrogen peroxide, where you can get at the ulcer and clean it without it. If you have a very great elevation of exuberant tissue, from granulation and fungation, you will find that an application of the nitrate of silver stick will often remove it in one day. It is remarkable how yielding it is to the application of the nitrate of silver stick. Where there is just an indolent sore with a slight pellicle over it and no effort to throw out a new epidermis from borders, I have used a ten per cent. application of nitrate of silver with extremely good results. You will find at first that there is a good deal of discharge and apparent aggravation, but you will find a little later that it has taken on healthy action. I have used various kinds of applications. In some of these cases where I had to use a dry powder on account of the discharge, I have used bismuth, and in some cases calomel. I have a prejudice against iodoform. I have seen bad effects from it very often. The next thing after you have got the ulcer thoroughly cleansed, is to simply apply an antiseptic gauze, preferably bichloride. The best method, I have found, though, was to use antiseptic or aseptic rubber tissue.

You take this rubber tissue cut into very narrow strips and lay it on over the wound, like the planks on a stable roof. This permits a little discharge. I have had a great deal of trouble in the treatment of wounds from the dressing sticking to the wound, and you frequently, in removing the dressing, tear the epidermis. The advantage of the rubber is that it does not stick to the wound, and I have never been able to take away other dressing without tearing the epidermis, more or less. As soon as scarification begins, you can frequently get a beautifully rapid healing by simply applying the rubber tissue directly to the wound, in these narrow strips. Sometimes I put in a little sterilized cotton first. In other cases I just put the rubber tissue directly on the wound. In other cases I put a little cotton or gauze, and then above that an adhesive strip.

In some cases where the healing has not gone along rapidly enough, I have found that an application of these narrow strips right over the sore is very good, and in that way you get a rapid disappearance of the fungating granulations. Of course, this is just a general idea of the matter. My idea in everything that I have said is to impress upon you my belief that it is not well to allow these sores to run on after they have done all that is necessary for the vaccination of the patient. I have found that if you can get the wound protected when it gets ready to begin skinning over, when the wound is, say one-fourth of an inch in diameter, you can get a rapid healing by just putting a little of the tissue over it to keep it from being rubbed.

In other cases, where it was necessary to turn the patients loose before they were entirely well, for instance a man going on a trip, I simply put on some common gauze and a few layers of the tissue over that, and then I usually found that the patient was well when I saw him again. It makes an uglier scar, from pressure, due to remaining on long, however, than it would otherwise. By the use of this rubber tissue, in the way I have explained, where you can keep the patient under your hands, when the epidermis becomes formed, you will find a very pretty scar.

### DISCUSSION.

*Dr. Crow:* I have not very much to say. I have been very much interested in Dr. Hutchins's talk, and think it is on the right line.

Every man, who is a general practitioner, has more or less of these old indolent sores from vaccination to treat, especially this last year. I concur in everything the doctor has said. I have not followed exactly the plan he has outlined in the treatment of these sores, but if a case should come under my observation now, I think I would do so, as it is probably a better plan than the one I used. Of course, in the first place, in the treatment of these indolent sores, you clean them up the best you can. My plan has been to use the borolyptol. I have a great deal of confidence in the formalin; I think there is two per cent. in it. Using that as a wash, I then use the powdered boracic acid and bismuth. I have followed the plan of leaving the wound open as far as possible where there is an abundance of secretion.

*Dr. Hancock:* In regard to vaccination, I have this last winter used the fluid vaccine—it comes in small tubes. Since I have used that I have made very little scarification usually, and I have had good success in taking. I never put but one scar on an arm. Some people vaccinate in three or four places. I think it is better to put but one. If you want to put two, put one on one arm and one on the other. In regard to these old sores, I have used burnt alum. Of course you call it a technical name when you put it on, but it works all right all the same. It works a good deal like nitrate of silver does.

*Dr. Hubbard:* I have been very much interested in the doctor's talk, because I have had some of these old sores run on for two or three months and kept me dressing them, and ordinarily people don't want to pay for it. I agree with him that it is a mixed infection. I have found that this bothers me most. I have used carbolic acid and cauterized the place. I have found that these old indolent sores occur usually in scrofulous or anemic persons. Take a healthy person and they are not usually troubled this way much; it is usually a better sore, but it generally heals up quickly. In these old sores, I have found that I got good results when I used carbolic acid or some cautery. As regards the use of gauze, I usually put it right down on the granulation. Of course, in some cases the granulation was below the surface, and then I used rubber tissue, as the doctor says, which I think is a fine thing. But I

don't use it as he does; I don't cut it in strips. It prevents the dressing from sticking, and that is a good deal gained. I have wondered why it was that some of them were so long healing. I couldn't account for it even on the ground of a mixed infection. I think thorough antisepsis is the plan, and I think it meets with success usually.

*Dr. Hood:* I want to thank Dr. Hutchins for the talk, and I think I will profit by the suggestions made. I want to report just one case of an old sore. I was called to see a patient who was suffering from pneumonia, and also from one of these old sores. She had been vaccinated, she said, in November, and the sore is not healed yet. She had neglected it—simply applied home remedies. I found under the sore a collection of pus. It was very soft and when I would press down the scab the pus would shoot out. I removed that scab and found dark looking granulation forming there. I have had three or four of these cases this winter, and have had some of them elevated above the skin a quarter of an inch. I took a pair of scissors and clipped this off and used pure sulphate of zinc, and then applied powdered boric acid, and they healed rapidly. That is almost the same as caustic. I thought about using nitrate of silver at the time, but I didn't have it, and I use the sulphate of zinc in its place and found it very successful. I had one case of a little boy who was vaccinated in December and had infection; he suffered, I think from septic infection and had eruptions all over his body. It had lasted several weeks—a month or six weeks. I had to sustain him with stimulants and combat the infection and finally he got well. I want to ask Dr. Hutchins his mode of vaccinating with liquor potassæ. I soon found that the quickest way I could adopt was to take a little wad of cotton and when I put it on to rub it off and do that frequently, and in about a minute I cleared away all the epidermis. I would apply it and wipe it off, apply it again and wipe it off. My experience with the ulcers is a good deal like the others' has been—mostly with children though, where they couldn't keep from scratching.

*Dr. Duncan:* As regards the liquor potassæ, I like it very much. I have been more successful in using it than in scarifying. I got a better idea partly from Dr. Hood and partly from failure to get good results by just putting it on the arm. In the way he describes you get results very quickly, in less than half the time it takes by just laying it on the arm. As regards the treatment of old sores, I am inclined to differ with Dr. Hutchins as to iodoform not being a good thing. I have never used it much, but I have used hydrozone or pyrozone, which is about the same thing. Then I put on a very small amount of sulphate of hydrastis and bismuth. One acting as an astringent and antiseptic, and the bismuth didn't amount to much, but it is a fine thing to dress a sore with. I think a great many of these sores are due to oleaginous applications. I am opposed to using any sort of oil or anything of that kind about the vaccination. I think those who have used no ointment at all have got along better than those who have used any ointment, no matter how antiseptic it was. I have made inquiry of those who came to me for treatment, and every one of them had been using some kind of ointment—had some sort of grease on it. Consequently I came to the conclusion that that had a great deal to do with keeping up the sore. My experience with the hydrozone in cleansing the arm and dressing it with bismuth—the next time time I have seen the patient I have found it much better. I have found that the patient would be better before he left my office—would say that his arm felt better. Next time I would see him, it would be very much better. Just a few dressings were required in all the cases I have seen.

*Dr. Hood:* I have had the arm colored to the end, so that it looked like erysipelas, and felt like it. Have you?

*Dr. Hutchins:* Yes.

*Dr. Childs:* There is one sort of ulcer I have had more trouble with than the kinds that have been mentioned. That is the deep ulcers in syphilitic cases. Two young men came to me. Both had been vaccinated, and had these deep ulcers. I treated them with iodide of potash. In regard to what Dr. Hancock says about vaccinating, I don't care to use two points on different arms. A man goes around with one arm in a sling; that is bad enough. He wouldn't look right with both arms in a sling. There is no use crippling a man on both arms. And as to vaccinating in several places, my teacher taught me this: Of those who were vaccinated with one point, ten per cent. had varioloid; of those vaccinated with two points, five per cent.; and of those vaccinated with four points, one-tenth of one per cent. I was in the city hospital in Charleston after I had been vaccinated. A young doctor friend who had been to the flower show came up to me—I won't call his name, he wasn't sober. He said: "Childs, by God, I have been to the flower show with the prettiest girl in South Carolina," and with that he came down on my arm and knocked the scab off and almost knocked my arm off. It was all I could do to keep from knocking him down. He didn't know it was sore though, I suppose; he didn't know anything, he was too drunk. That made it a long time getting well. In regard to these deep syphilitic ulcers, I think it is important to keep the arm in a sling.

*Dr. Hancock:* I didn't intend to convey the idea that I vaccinated on more than one arm as a rule, but there are some cases in which that has happened to me. A person with the smallpox came in my office one day, and my driver had never been successfully vaccinated. I vaccinated him on both arms and it took very well on both of them.

*Dr. Champion:* I haven't anything to say except that I agree with Dr. Duncan. I never use ointment of any kind on a sore. I think grease in a sore anywhere on the body is a receptacle for germs. The only place I use grease is in passing a sound in the penis.

*Dr. Hutchins:* I want to thank you, gentlemen, for the way in which you have have received my talk. I have tried to impress on the students in the college not to know formula, but that they ought to know principles. That is the way with these sores. Just whatever you find is best, that is the thing to use. Now as to what Dr. Hancock says about the number of vaccinations. Some time ago, Dr. A. W. Stirling, who was educated in Edinburgh, I believe, published a paper in which he stated figures something like the figures given by Dr. Childs. I have believed in multiple vaccination since that time. About what Dr. Hubbard says about the sores taking so long to get well; I suspect they were worse cases than I saw. My cases averaged about ten days to two weeks in getting well under the treatment I used. About the use by Dr. Hood of my liquor potassæ method, I have learned something to-night. By putting it on and rubbing it off he gets rid of that soapy product which is caused by the fatty secretion of the skin uniting with the liquor potassæ. That is a very valuable point. Now as to this peroxide of hydrogen; that is my own notion. I have seen unsatisfactory results from it, and I don't like to use it. About Dr. Childs's syphilitic vaccine sores; I have not seen any of them. The idea with me would be to follow the same principles, but I would use some form of mercury on these sores. My experience has been that it is best to use mercury locally on syphilitic sores. I agree with the gentlemen fully about ointment not being good for sores, but if you have an ointment that is sufficiently antiseptic, it will frequently do what the rubber tissue does, and you can use it on the face and elsewhere where you cannot use the rubber tissue, and it will do the work very well indeed. In treating these syphilitic sores, I would not hesitate to use ordinary mercurial ointment, either alone or with other things, and I think you will find that you will get a good effect from it.

*Dr. Childs:* I have used the white precipitate ointment on these sores with good results. I can't concur that all ointments are bad. There is not anything that gives better results in some cases than boracic acid ointment. I am a great believer in boracic acid ointment, and I use white precipitate ointment on these syphilitic sores.

---
